# Hydrophobic silver nanoparticles trapped in lipid bilayers: Size distribution, bilayer phase behavior, and optical properties

**DOI:** 10.1186/1477-3155-6-13

**Published:** 2008-11-12

**Authors:** Geoffrey D Bothun

**Affiliations:** 1 Department of Chemical Engineering, University of Rhode Island, Kingston, RI, 02881, USA

## Abstract

**Background:**

Lipid-based dispersion of nanoparticles provides a biologically inspired route to designing therapeutic agents and a means of reducing nanoparticle toxicity. Little is currently known on how the presence of nanoparticles influences lipid vesicle stability and bilayer phase behavior. In this work, the formation of aqueous lipid/nanoparticle assemblies (LNAs) consisting of hydrophobic silver-decanethiol particles (5.7 ± 1.8 nm) embedded within 1,2-dipalmitoyl-sn-glycero-3-phosphocholine (DPPC) bilayers is demonstrated as a function of the DPPC/Ag nanoparticle (AgNP) ratio. The effect of nanoparticle loading on the size distribution, bilayer phase behavior, and bilayer fluidity is determined. Concomitantly, the effect of bilayer incorporation on the optical properties of the AgNPs is also examined.

**Results:**

The dispersions were stable at 50°C where the bilayers existed in a liquid crystalline state, but phase separated at 25°C where the bilayers were in a gel state, consistent with vesicle aggregation below the lipid melting temperature. Formation of bilayer-embedded nanoparticles was confirmed by differential scanning calorimetry and fluorescence anisotropy, where increasing nanoparticle concentration suppressed the lipid pretransition temperature, reduced the melting temperature, and disrupted gel phase bilayers. The characteristic surface plasmon resonance (SPR) wavelength of the embedded nanoparticles was independent of the bilayer phase; however, the SPR absorbance was dependent on vesicle aggregation.

**Conclusion:**

These results suggest that lipid bilayers can distort to accommodate large hydrophobic nanoparticles, relative to the thickness of the bilayer, and may provide insight into nanoparticle/biomembrane interactions and the design of multifunctional liposomal carriers.

## Background

Hybrid lipid/nanoparticle conjugates provide a biologically inspired means of designing stable agents for biomedical imaging, drug delivery, targeted therapy, and biosensing [1]. An advantage of using lipids as stabilizing or functional ligands is that they mimic the lipidic scaffolding of biological membranes and have well-characterized physicochemical properties and phase behavior. In lipid vesicles, nanoparticle encapsulation can be achieved by trapping particles within the aqueous vesicle core or within the hydrophobic lipid bilayer. Becker et al [2], Kim et al [3], and Zhang et al [4] have shown that iron oxide (Fe_3_O_4_), cadmium selenide (CdSe) quantum dots, and gold nanoparticles, respectively, can be trapped within aqueous vesicle cores. To embed nanoparticles within lipid bilayers, the nanoparticle must be small enough to fit within a DPPC bilayer and it must present a hydrophobic surface. Using physisorbed stearylamine, Park et al [5,6] have stabilized 3–4 nm gold and silver particles in 1,2-dipalmitoyl-sn-glycero-3-phosphocholine (DPPC) bilayers. Likewise, Jang et al [7] embedded 2.5–3.5 nm silicon particles with chemisorbed 1-octanol into bilayer membranes composed of DOXYL-labeled phosphocholine lipids. The resulting vesicles are analogous to liposomal drug delivery systems with an added functional nanoparticle component.

For hydrophobic nanoparticles embedded within lipid bilayers, which is the focus of this work, the presence of nanoparticles can lead to changes in lipid packing and may disrupt lipid-lipid interactions amongst the headgroups and/or acyl tails [5,6]. Disruption of such inter-lipid interactions can result in changes in lipid bilayer phase behavior, which is related to the degree of lipid ordering and bilayer viscosity. Hence, depending on their size and surface chemistry, embedded nanoparticles may influence the stability and function of hybrid vesicles, as well as the conditions required for preparation.

**Figure 1:**
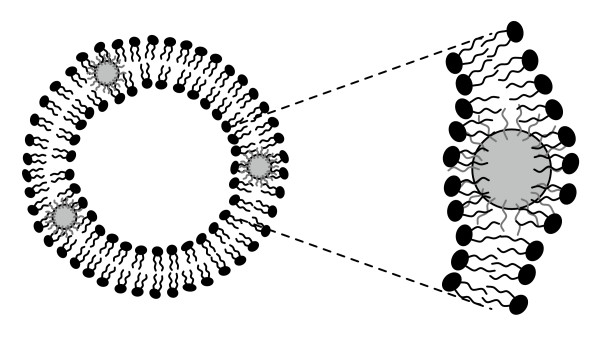
**A lipid/nanoparticle assembly (LNA) containing hydrophobic nanoparticles embedded within vesicle bilayers**. This illustration, which was adapted from Jang et al [7], depicts the incorporation of nanoparticles that have been surface modified with hydrophobic tails (e.g. decanethiol, shown in gray) into a lipid bilayer. Lipid disordering and bilayer disruption will be dependent on the size and surface chemistry of the nanoparticles. The image is not to scale.

This work demonstrates the formation of hybrid lipid/nanoparticle assemblies (LNAs) containing hydrophobic decanethiol-modified silver nanoparticles (Ag-decanethiol) and the effect of embedded nanoparticles on bilayer structure. An illustration of a vesicle assembly is shown in Figure 1 (not to scale). DPPC, a zwitterionic phospholipid with dual saturated C_16 _tails, was chosen for this study as a model lipid system because of its well-characterized phase behavior [8]. Vesicle size, stability, and bilayer phase behavior were examined as a function of nanoparticle loading and temperature. Ag LNAs were also formed with a mixture of DPPC and 1,2-dipalmitoyl-sn-glycero-3- [phospho-L-serine] (DPPS), an anionic phospholipid, to investigate the effect of vesicle charge and aggregation on the Ag SPR wavelength.

## Methods

### Chemicals

**Figure 2:**
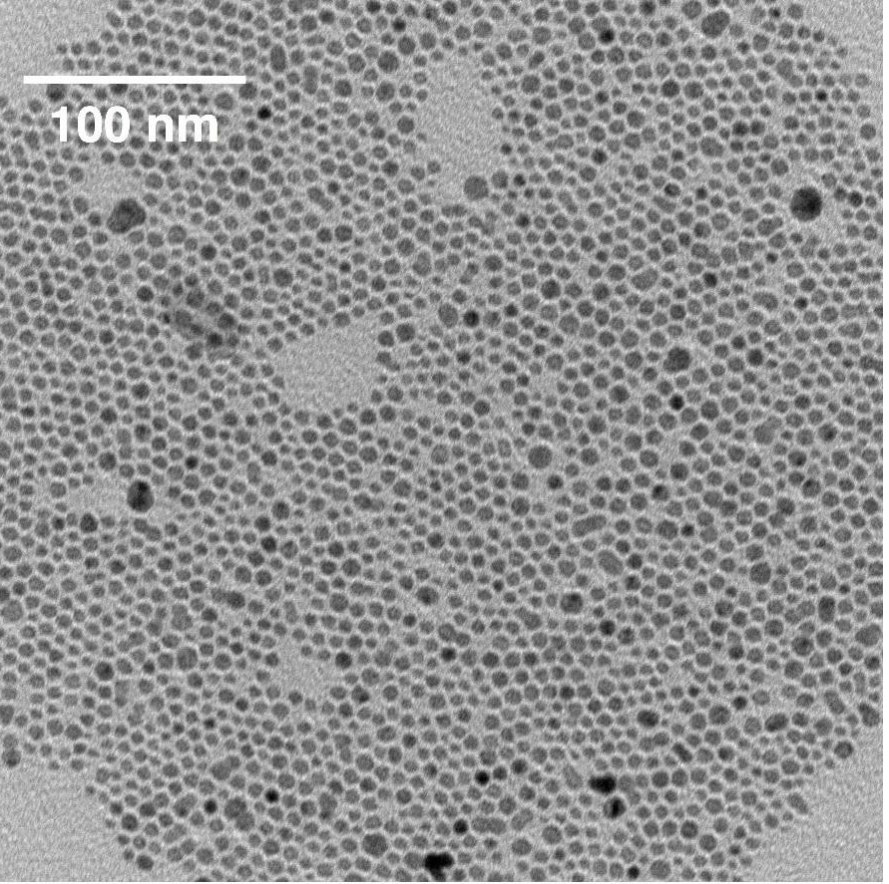
**Size distribution of Ag-decanethiol nanoparticles**. An aliquot of the AgNPs in hexane was dried on a lacy carbon grid and images were taken using a transmission electron microscope. The average nanoparticle diameter was determined by ImageJ analysis software [9].

DPPC and DPPS (>99%) were obtained from Avanti Polar Lipids, and chloroform and tetrahydrafuran (THF) from Fisher Scientific (>99.9%). Diphenylhexatriene (DPH) and Ag-decanethiol nanoparticles (AgNPs) dispersed in hexane (0.1 wt%) were obtained from Sigma-Aldrich. An average nanoparticle diameter of 5.7 ± 1.8 nm was measured by transmission electron microscopy (JOEL JEM 1200EX) using ImageJ analysis software [9] (Figure 2). Dulbecco's 150 mM phosphate buffered saline (PBS) was prepared at pH 7.4 with sterile deionized water from a Millipore Direct-Q3 UV purification system.

### DPPC/AgNP and DPPC/DPPS/AgNP assembly formation

Lipid assemblies were prepared in PBS at 1 and 30 mM DPPC using the Bangham method [10]. The 1 mM DPPC samples were prepared for fluorescence anisotropy measurements using DPH as a bilayer probe molecule. In these samples, the AgNP concentration was varied from 1 to 1000 mg/L to provide DPPC/AgNP ratios from 734:1 to 1:1 (w/w), respectively. The 30 mM DPPC samples were prepared for differential scanning calorimetry (DSC) and dynamic light scattering (DLS) studies. For these samples, the AgNP concentration was varied from 0.1 to 11.0 g/L to provide DPPC/AgNP ratios from 200:1 to 2:1 (w/w). To form the LNAs, an aliquot of the Ag-decanethiol NP/hexane solution was added to DPPC dissolved in chloroform to yield a transparent, miscible brown phase. For anisotropy measurements, an aliquot of DPH in THF was also added at a DPPC to DPH molar ratio of 500:1. The solvent phase was evaporated under nitrogen and the sample was placed under vacuum for 2 hours, leaving a dry DPPC/AgNP film. Hydration and processing steps were performed at 50°C, which is above the DPPC gel-fluid melting temperature (*T*_*m *_= 42°C). The films were hydrated with PBS, incubated for 1 hour, and sonicated for 2 hours. Portions of each sample were stored at 25°C (gel phase bilayers) and 50°C (fluid phase bilayers) for 15 days without agitation.

LNAs were also prepared with a lipid mixture of DPPC and DPPS at a molar ratio of 85:15, and a lipid/AgNP weight ratio of 100:1. In this case DPPS was dissolved in a 1:2 chloroform to methanol mixture, and added to the DPPC/chloroform + AgNP/hexane solution. The melting temperature of the mixed DPPC/DPPS bilayer without AgNPs was 43.4°C (measured by DSC).

### Colloidal stability and size distribution: Dynamic light scattering

The hydrodynamic diameter and stability of the assemblies were analyzed at the storage temperatures (25 or 50°C) using a Brookhaven light scattering system consisting of a BI-200SM goniometer, a Lexel 95-2 argon laser, and a BI-9000AT Digital Correlator. DLS samples were analyzed at 0.4 mM DPPC. Size distributions were obtained using a continuous non-negative least squares (NNLS) fit of the autocorrelation function (*RMS *< 3.6 × 10^-3^).

### Bilayer phase behavior: Differential scanning calorimetry

The pretransition temperatures associated with gel to rippled-gel lipid bilayer transitions, and the main transition or melting temperatures associated with rippled-gel to fluid transitions, were analyzed by differential scanning calorimetry (DSC, TA Instruments Q10) at 30 mM DPPC. Heat/cool scans were conducted from 25 to 50°C at 1°C/min.

### Bilayer melting and fluidity: Fluorescence anisotropy

Bilayer melting temperatures and fluidity were also examined by fluorescence anisotropy (Perkin Elmer LS 55) of the hydrophobic bilayer probe diphenylhexatriene (DPH) at 1 μM DPPC from 30 to 50°C at a rate of 1°C/min under continuous mixing. Steady-state DPH anisotropy within the DPPC bilayer was determined at *λ*_*ex *_= 350 nm and *λ*_*em *_= 452 nm using the expression <*r*> = (*I*_*VV *_- *I*_*VH*_)/(*I*_*VV *_+ *GI*_*VH*_) where *I *represents the fluorescence emission intensity, *V *and *H *represent the vertical and horizontal orientation of the excitation and emission polarizers, and *G *= *I*_*HV*_/*I*_*HH *_accounts for the sensitivity of the instrument towards vertically and horizontally polarized light [11].

### Optical properties: Ultraviolet-visible (UV-vis) spectroscopy

The optical absorbance properties of DPPC/AgNP vesicles were examined by UV-vis spectroscopy (Varian Cary 50) at 0.6 mM DPPC from 25 to 55°C under mixing. For varying DPPC/AgNP ratios, the absorbance data presented was normalized against the absorbance at 300 nm (*A*/*A*_300_) to account for differences in turbidity amongst the samples. Raw absorbance data is presented for fixed DPPC/AgNP and DPPC/DPPS/AgNP ratios.

## Results and discussion

### Synthesis and stability of hybrid DPPC/AgNP assemblies

**Figure 3:**
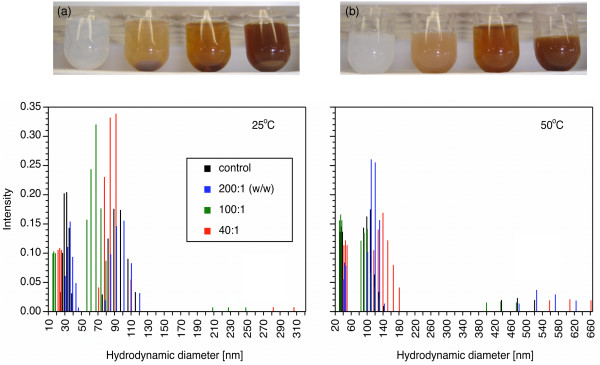
**Colloidal stability and size distribution of DPPC vesicles as a function of Ag-decanethiol nanoparticle loading and storage temperature**. The stability of DPPC/AgNP vesicles prepared at 30 mM DPPC in PBS is shown after 15 days at storage temperatures below (top a, 25°C) or above (top b, 50°C) the main phase transition temperature. From left to right, the samples correspond to the control, 200:1, 100:1, and 40:1 DPPC/AgNP (w/w). Size distributions determined by dynamic light scattering (DLS) are shown for the corresponding conditions (bottom).

For samples prepared at 30 mM DPPC, an increase in AgNP loading from DPPC/AgNP ratios of 200:1 to 40:1 (w/w) caused the sample color to change from a pale to dark reddish brown color (Figure 3). Samples maintained at 25°C phase separated to form a settled layer (Figure 3a) while samples maintained at 50°C remained dispersed for 15 days (Figure 3b). Phase separation at 25°C was attributed to the agglomeration, fusion, and sedimentation of DPPC vesicles, which is greater in gel phase bilayer vesicles than fluid phase [12]. Size distribution measurements using DLS showed that the top phase of the samples stored at 25°C, below *T*_*m*_, had a size distribution that included two dominant fractions between 15 and 46 nm and 56 and 120 nm. The size distribution and extent of sonication during sample preparation [13] are consistent with small unilamellar vesicles (SUVs). When stored a 50°C, three fractions were observed between 30 and 49 nm, 84 and 180 nm, and 399 and 661 nm. In addition to unilamellar vesicles, unilamellar agglomerates were observed by light microscopy (100× oil-immersion lens; images not shown). The presence of nanoparticles did not significantly affect the size distributions at either temperature.

### Phase behavior and fluidity of DPPC/AgNP bilayers

**Figure 4:**
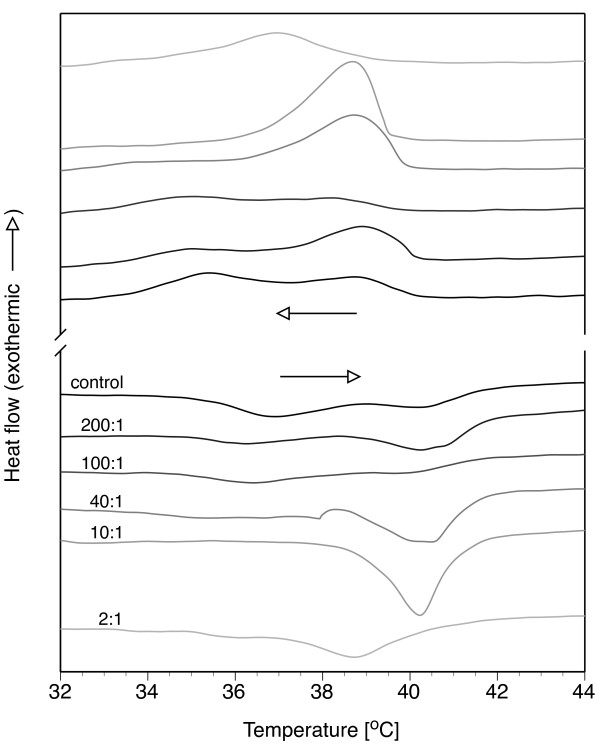
**Lipid phase behavior as a function nanoparticle loading determined by calorimetry**. Lipid bilayer phase behavior of DPPC/AgNP vesicles (30 mM DPPC) was determined by differential scanning calorimetry (DSC) for a single heat/cool cycle at 1°C/min. The tilted gel to rippled gel pre-transitions and the rippled gel to fluid main transitions (melting) are visible. The pretransition and melting temperatures were taken at the point of maximum heat flow.

Changes in the pretransition and melting temperatures of DPPC/AgNP bilayers formed at 30 mM DPPC were examined by DSC. For the control sample, the pretransition and melting regions overlapped and had maximum heat flows at 36.9 and 40.4°C, respectively (Figure 4). These values are consistent with SUV DPPC vesicles prepared by ultrasonication, which exhibit broad melting regions due to constraints imposed on the lipid molecules by the small radii of curvature relative to large unilamellar or multilamellar vesicles [14,15]. Sequential heating and cooling curves indicated that the pretransition of DPPC was influenced by the presence of the AgNPs, while the melting temperature (*T*_*m*_) was less sensitive from 200:1 to 10:1 DPPC/AgNP (Figure 4). When compared to the control, a DPPC/AgNP ratio of 40:1 reduced the pretransition temperature by 1.8°C, yet had no effect on *T*_*m*_. The pretransition was not observed during heating or cooling at high AgNP concentrations of 10:1 and 2:1. At 2:1, the melting was reduced to 38.8°C, which is a 1.6°C reduction relative to the control. DSC results are summarized in Table 1.

**Table 1:** Phase transition temperatures of DPPC/AgNP assemblies determined by DSC.

DPPC/AgNP	Pretransition^a^	Melting^a^
[w/w]	[°C]	[°C]
control	36.9	40.4
200:1	36.3	40.3
100:1	36.4	40.1
40:1	35.1	40.5
10:1	-	40.2
2:1	-	38.8

^a^Increasing temperature run. Taken at the temperature corresponding with the maximum heat flow.

**Figure 5:**
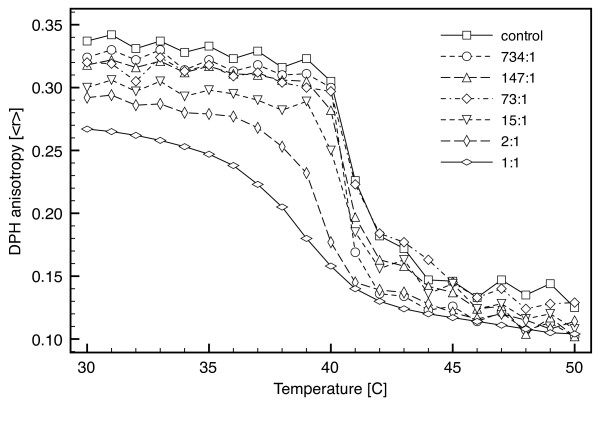
**Bilayer fluidity and melting as a function nanoparticle loading determined by fluorescence anisotropy**. Fluorescence anisotropy of diphenylhexatriene in DPPC bilayers was measured as a function of the DPPC/AgNP weight ratio and temperature (1°C/min). DPPC/AgNP samples prepared at 1 mM were diluted 1000-fold for analysis. Anisotropy, <*r*>, is a measure of lipid ordering and the bilayer microviscosity. Gel phase bilayers exhibit high anisotropy and fluid phase bilayers exhibit low anisotropy. The transition from high to low anisotropy with increasing temperature denotes the gel to fluid melting transition. The midpoint of the transition is taken as the melting temperature.

To confirm DSC results and measure bilayer fluidity in the gel and fluid phases, 1 mM samples were prepared at similar DPPC/AgNP weight ratios and diluted to 1 μM DPPC for fluorescence anisotropy measurements. DSC directly measures the enthalpy associated with a gel to fluid transition, while fluorescence anisotropy measures the anisotropy of DPH (aligned parallel to the lipid tails) due to changes in the degree of lipid ordering. Lipid ordering is related to the microviscosity, which is higher in the gel phase than the fluid phase. From 734:1 to 73:1 DPPC/AgNP, which corresponded to 1 to 10 mg AgNP/L, the presence of nanoparticles had little affect on the melting temperature and temperature range relative to the control (Figure 5). The control melted at ca. 41°C over a temperature range (Δ*T*_*m*, *r*_) of 2°C. However, there is a decrease in the melting temperature at DPPC/AgNP ratios less than 15:1, or above 50 mg AgNP/L. This decrease is appreciable at 2:1 (*T*_*m *_≈ 39.5°C; Δ*T*_*m*, *r *_≈ 5°C) and 1:1 (*T*_*m *_≈ 38.5°C; Δ*T*_*m*, *r *_≈ 7°C). The reduction in *T*_*m *_relative to the control measured by fluorescence anisotropy is in agreement with the DSC results.

In addition to affecting the melting temperature, the AgNPs increased bilayer fluidity (i.e. reduced lipid ordering) of the gel phase (Table 2). For instance, at 30°C in gel phase bilayers with a high degree of lipid ordering, DPH anisotropy (<*r*>) decreased from 0.337 to 0.267 at DPPC/AgNP ratios of 734:1 and 1:1, respectively. At 50°C in fluid phase bilayers, a decrease from 0.125 to 0.104 was also observed at the same nanoparticle loadings. Anisotropy results for the gel phase indicate appreciable fluidization that was not observed in the aforementioned study by Park et al [5]. However, in their work the AgNPs were smaller (3–4 nm), stabilized by physisorbed stearylamine, and had little affect on gel phase fluidity. The results obtained in this work suggest that larger particles stabilized by decanethiol promote lipid disordering.

**Table 2:** Melting temperature and bilayer fluidity determined by fluorescence anisotropy of diphenylhexatriene (DPH).

DPPC/AgNP		<*r*>^a^		Melting^a, b^
[w/w]	mg AgNP/L	30°C	50°C	[°C]
control	0	0.337	0.125	41.0
734:1	1	0.324	0.102	40.7
147:1	5	0.318	0.102	40.7
73:1	10	0.320	0.129	41.0
15:1	50	0.300	0.108	40.5
2:1	500	0.292	0.114	39.5
1:1	1000	0.267	0.104	39.0

^a^Increasing temperature run.

^b^Determined graphically from the transition midpoint.

DSC and fluorescence anisotropy results indicate that the hydrophobic nanoparticles were interacting with the bilayer in a concentration-dependent manner. Given the hydrophobicity of the nanoparticles and their preference to partition into a hydrophobic environment, it is likely that a portion or all of the nanoparticles were embedded within the bilayer acyl region (Figure 1) and suppressed the pretransition and melting temperatures via bilayer disruption. The pretransition involves the transformation of a tilted-gel phase to a more disordered rippled-gel phase. While the rippled-gel phase is not completely understood, it has been described as being a gel phase that contains liquid crystalline domains [16]. Mismatches between the bilayer thickness of neighboring gel and liquid crystalline phases produce periodic ripples. The absence of a pretransition with increased AgNP loading suggests that the presence of the nanoparticles inhibited ripple formation. Bilayer melting describes the transition from a rippled-gel to liquid crystalline phase, or fluid phase, due to melting of the lipid acyl tails. The highest nanoparticle loadings (2:1 and 1:1) suggest that the bilayer was appreciably disrupted by the presence of the nanoparticles.

Bilayer disruption was demonstrated; however, nanoparticle-lipid interaction mechanisms, as well as the structure and morphology of the LNAs are still under investigation. It is likely that the smaller nanoparticles in the size distribution embedded within the bilayers, while the larger particles were capped and dispersed in the aqueous phase by a lipid monolayer with the C_16 _acyl tails mixing with the decanethiol tails and the headgroups exposed to water. Lipid-capped nanoparticles and possible agglomerates are consistent with the smaller size fractions measured by DLS. Previous experimental studies have been focused on nanoparticle diameters smaller than 5 nm, which is a typical thickness for a lipid bilayer [5,6,17]. However, recent computer simulations suggest that it is thermodynamically feasible for 2–8 nm diameter nanoparticles to embed within a lipid bilayer [18]. Based on bilayer phase behavior, it is shown herein that it may be possible to embed nanoparticles that have a diameter in proximity to, or exceeding the thickness of the bilayer, which is consistent with the simulation work [18].

### Optical properties of DPPC/AgNP and DPPC/DPPS/AgNP vesicles

**Figure 6:**
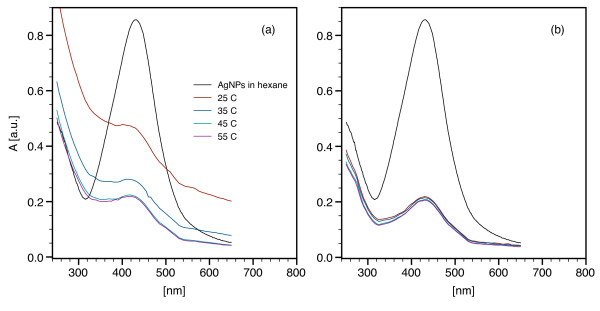
**Surface plasmon resonance (SPR) of lipid/Ag-decanethiol nanoparticle assemblies as a function of temperature**. UV-vis spectroscopy was used to confirm the native AgNP SPR peak in hexane and in lipid/AgNP vesicle suspensions (0.6 mM lipid; 100:1 w/w). Optical properties of (a) DPPC/AgNP and (b) DPPC/DPPS/AgNP vesicles are shown as a function of temperature, which span the gel and fluid bilayer phases.

**Figure 7:**
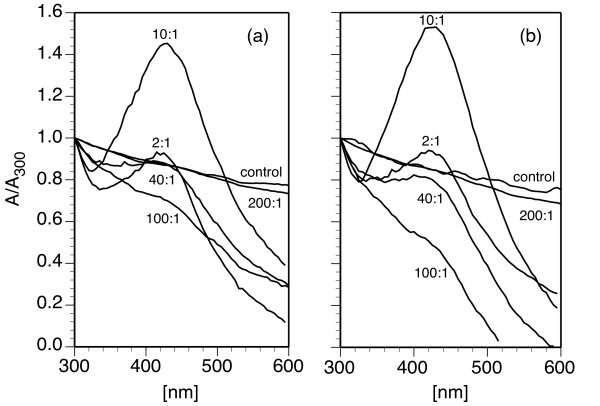
**Surface plasmon resonance (SPR) of DPPC/Ag-decanethiol nanoparticle assemblies as a function of nanoparticle loading**. The AgNP SPR peak was used to confirm was measured by UV-vis spectroscopy in DPPC/AgNP vesicle suspensions (0.6 mM DPPC) at (a) 25 and (b) 50°C as a function of the DPPC/AgNP ratio, 200:1 to 2:1 (w/w). These temperatures correspond to gel and fluid phase bilayers, respectively. Normalized absorbance values are presented relative to *λ*_300 nm_.

Native AgNPs dispersed in hexane exhibited a reddish brown color and a SPR peak at 430 nm (Figure 6). When dispersed as DPPC/AgNP vesicles at 100:1, the SPR wavelength was not influenced by lipid encapsulation or temperature (Figure 6a). At 25°C, below *T*_*m*_, gel phase DPPC/AgNP vesicles yielded a more turbid sample and a higher absorbance due to aggregation, which is in agreement with the DLS results. The absorbance is lower at 35°C, which is near the rippled-gel transition and inhibits aggregation relative to 25°C. At 45 and 55°C, above *T*_*m*_, the absorbance spectra for fluid phase vesicles were consistent with a less aggregated, and hence less turbid, sample. For all DPPC/AgNP weight ratios, AgNP SPR peaks were observed from 425 to 430 nm and further verified the incorporation of AgNPs within the suspensions (Figure 7).

DPPC/DPPS/AgNP assemblies (85:15 DPPC to DPPS) were prepared at 100:1 lipid/AgNP to further investigate the effect of aggregation. DPPS is an anionic lipid that stabilizes vesicles via electrostatic repulsion. With the addition of DPPS, there was no change in the SPR wavelength relative to the native AgNPs in hexane or DPPC/Ag vesicles. DPPC/DPPS/AgNP vesicles remained stable and the absorbance spectra were similar for both the gel and fluid phase (Figure 6b). Results for both the zwitterionic and mixed zwitterionic/anionic lipids suggest that neither AgNP encapsulation within the bilayers or vesicle aggregation affect the SPR wavelength, as AgNP aggregation has been shown to yield a prominent red-shift [19].

Comparatively, Bhattacharya and Sirvastava [20] have shown that 2.04 ± 0.4 nm gold nanoparticles containing a hydrophobic surface ligand maintain their characteristic SPR band when embedded within gel phase DPPC bilayers. This work expands upon this observation, and suggests that the SPR of small AgNPs was independent of bilayer phase at the DPPC/AgNP and DPPC/DPPS/AgNP ratios examined.

## Conclusion

Aqueous dispersions of hydrophobic Ag-decanethiol nanoparticles were formed using DPPC and DPPC+DPPS as stabilizing components. Our results based on bilayer phase behavior suggest that the DPPC/AgNP assemblies consisted of nanoparticle-embedded bilayer vesicles. The stability of the assemblies was dependent on their storage temperature and, in turn, the state of the bilayer (gel or fluid phase). Given that the nanoparticles had diameters near or exceeding the thickness of a lipid bilayer, this work suggests that DPPC bilayers can distort to accommodate such particles and that this distortion reduces lipid ordering. This result is consistent with the ability for a cell membrane to accommodate large transmembrane proteins [21]. As a therapeutic agent, LNAs may be formed with functional nanoparticles, potentially larger than previously thought, for combined delivery and imaging. With respect to nanoparticle-cell interactions, these results provide further evidence that such hydrophobic nanoparticles could reside within cell membranes. Studies are underway to measure LNA morphology and structure, develop new nanoparticle encapsulation protocols, and explore different lipid compositions.

## Competing interests

The author declares that he has no competing interests.
